# Supplementary material for: Sustainable upcycling of mixed spent cathodes to a high-voltage polyanionic cathode material
Source: Nat Commun. 2024 May 14;15:4086. doi: 10.1038/s41467-024-48181-9 (PMC11094161; doi:10.1038/s41467-024-48181-9)
Supplement: Supplementary file 1 — Supplementary Information [file 41467_2024_48181_MOESM1_ESM.pdf]

Supplementary Information for

Sustainable upcycling of mixed spent cathodes to a high-voltage polyanionic cathode material

Guanjun Ji[1,2#], Di Tang[1#], Junxiong Wang[1,2*], Zheng Liang[2], Haocheng Ji[1], Jun Ma[1], Zhaofeng Zhuang[1], Song Liu[1], Guangmin Zhou[1*], Hui-Ming Cheng[3,4*]

[1] *Tsinghua Shenzhen International Graduate School, Tsinghua University, Shenzhen 518055, China*

[2] *Frontiers Science Center for Transformative Molecules, School of Chemistry and Chemical Engineering, Shanghai Jiao Tong University, Shanghai 200240, China*

[3] *Institute of Technology for Carbon Neutrality / Faculty of Materials Science and Engineering, Shenzhen Institute of Advanced Technology, Chinese Academy of Science, Shenzhen 518055, China*

[4] *Shenyang National Laboratory for Materials Science, Institute of Metal Research, Chinese Academy of Sciences, Shenyang 110016, China*

* Corresponding authors: Junxiong Wang (wjx1992@sjtu.edu.cn); Guangmin Zhou (Email: guangminzhou@sz.tsinghua.edu.cn); Hui-Ming Cheng (Email: hm.cheng@siat.ac.cn)

# These authors contributed equally to this work.

This file includes:

Supplementary Text

Supplementary Fig. 1 to Fig. 34

Supplementary Table 1 to Table 12

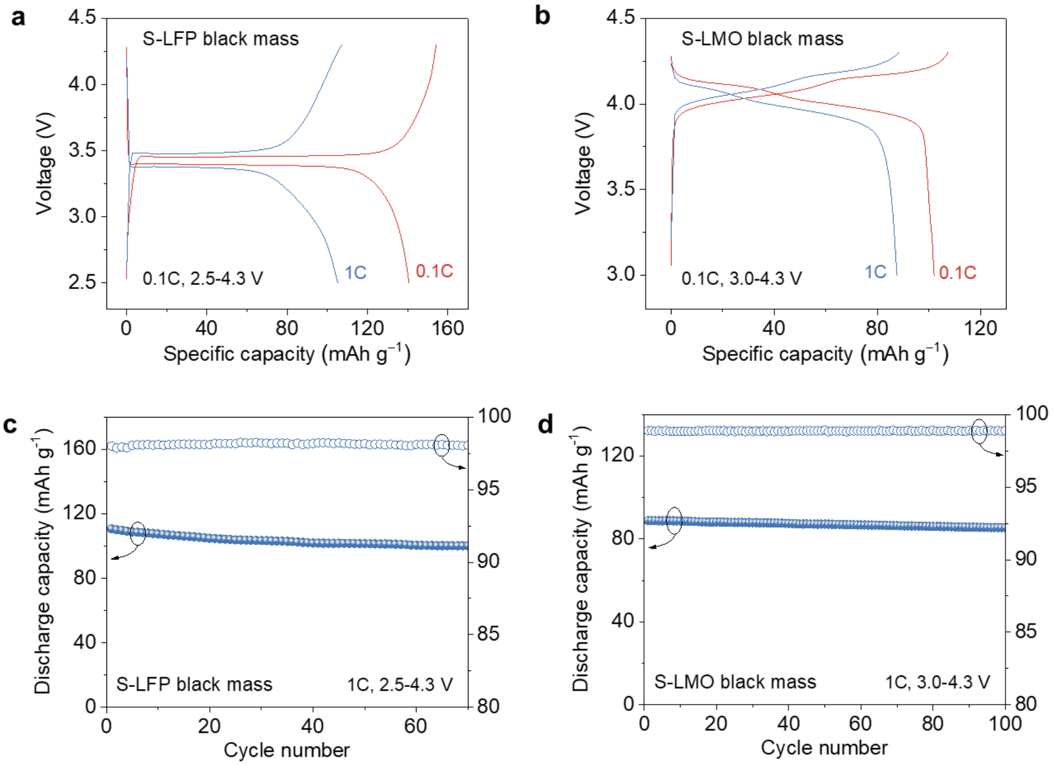

Supplementary Fig. 1 Electrochemical performance of S-LFP and S-LMO black mass used in this work. **a, b** Charge and discharge curves of **a** S-LFP and **b** L-LMO. **c, d** Cycling performance of **c** S-LFP and **d** L-LMO.

To reveal the state of health, half cells were assembled to measure the electrochemical performance. S-LFP have specific capacities of 140 and 110 mAh g^{-1} at 0.1 and 1C, respectively. Lithium loss, resulting in the formation of a Fe(III) phase, is the main reason for the capacity degradation. For S-LMO, the discharge capacities are 110 and 90 mAh g^{-1} at 0.1 and 1C, respectively.

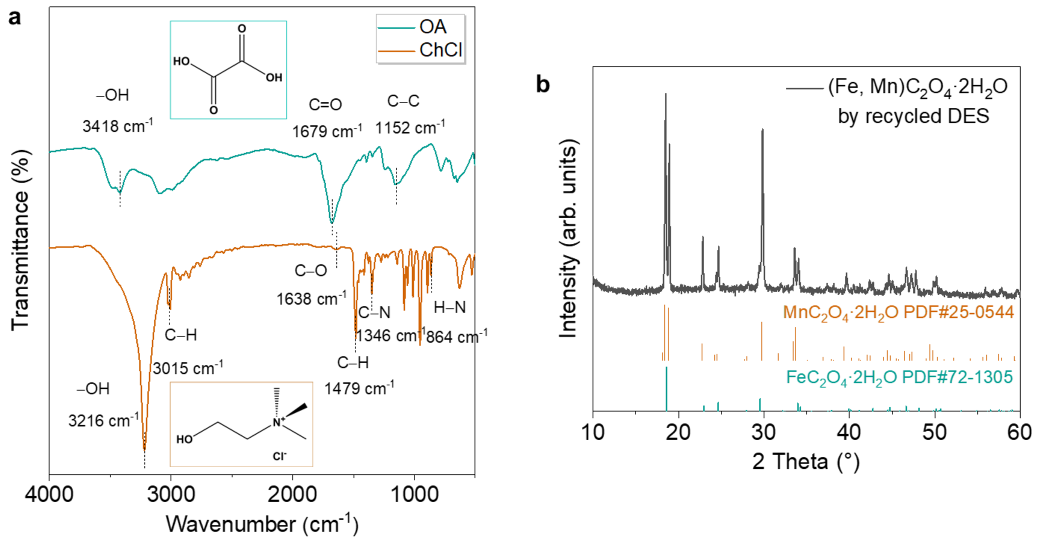

Supplementary Fig. 2 Structural characterization for ChCl, OA and the precursor. **a** FT-IR spectra of ChCl and OA. **b** XRD pattern of (Fe, Mn)$C_2O_4 \cdot 2H_2O$ precursor formed using the recycled DES.

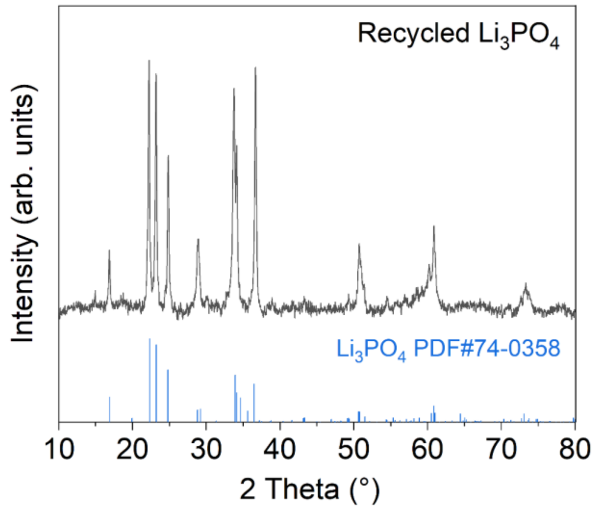

Supplementary Fig. 3 XRD pattern of the recycled Li_3PO_4.

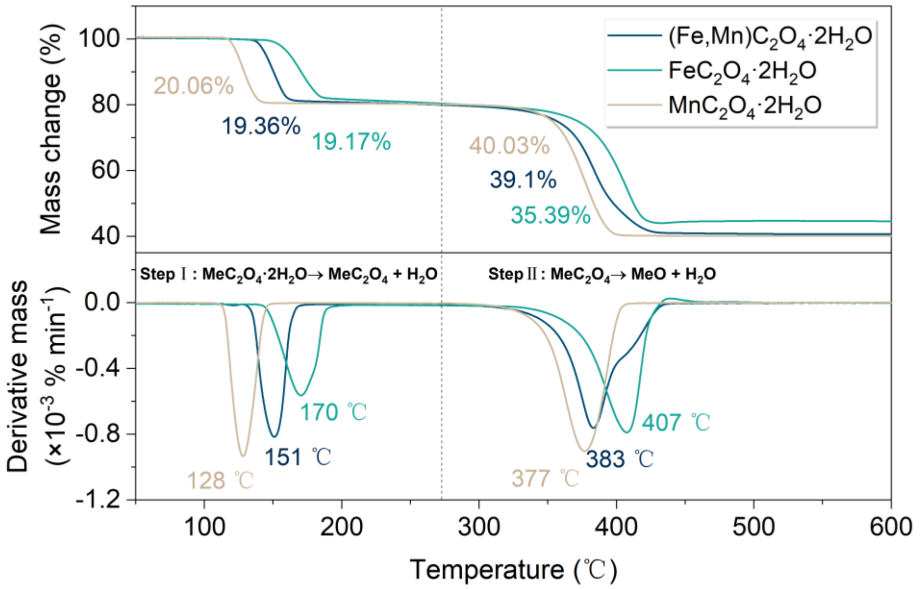

Supplementary Fig. 4 TGA of $FeC_2O_4 \cdot 2H_2O$, $MnC_2O_4 \cdot 2H_2O$, and (Fe, Mn)$C_2O_4 \cdot 2H_2O$ precursor.

In general, thermal decomposition of a single transition-metal oxalate has two steps of mass loss. One is the dehydration of the oxalate at a low temperature, and the other is the decomposition of the dehydrated oxalate at a high temperature[1]. The onset decomposition temperature of the oxalate is closely related to the electronegativity of the centering metal ions in the coordination compound of the oxalates[2, 3]. As a result, the mass change of a mixture of two oxalates generally shows four steps. However, there are only two steps of mass loss in the (Fe, Mn)$C_2O_4 \cdot 2H_2O$ precursor, confirming it is a solid solution.

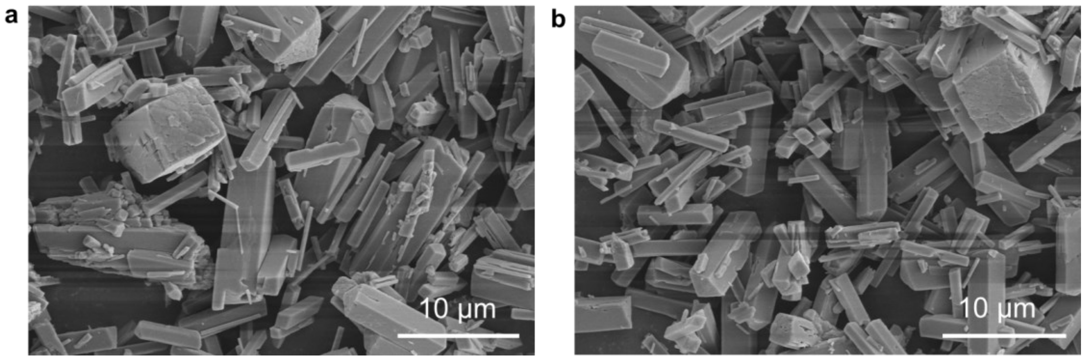

Supplementary Fig. 5 SEM images of the (Fe, Mn)$C_2O_4 \cdot 2H_2O$ precursor (scale bar, 10 μm).

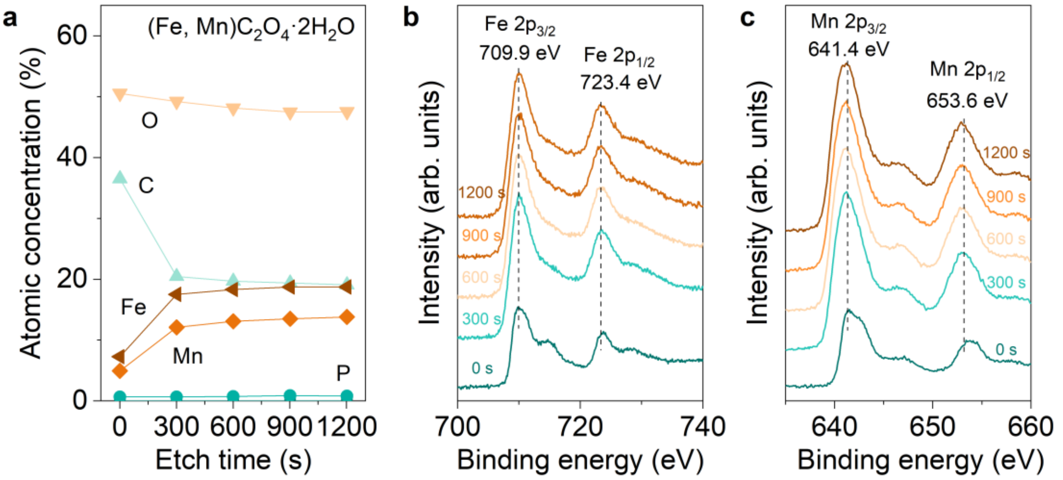

Supplementary Fig. 6 Depth etching XPS results of the (Fe, Mn)$C_2O_4 \cdot 2H_2O$ precursor. **a** Atomic concentrations. **b** Fe 2*p* XPS spectra. **c** Mn 2*p* XPS spectra.

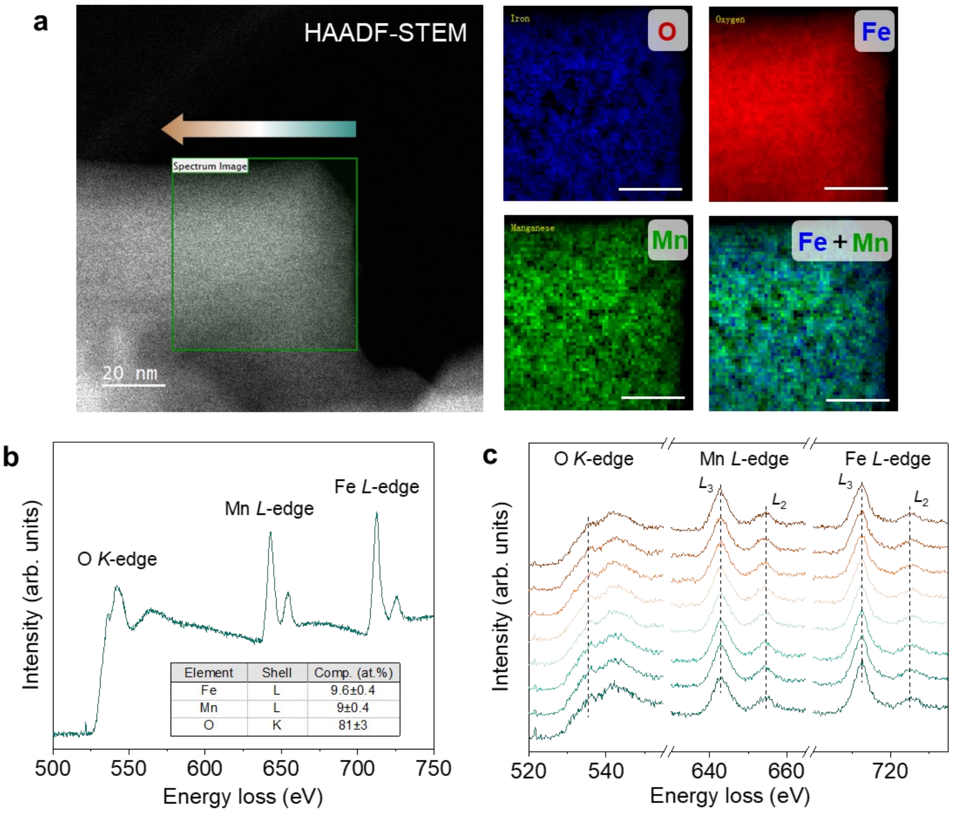

Supplementary Fig. 7 Microstructure and element distribution of (Fe, Mn)$C_2O_4 \cdot 2H_2O$ precursor. **a** HAADF-STEM image and O, Fe, Mn, and mixed element maps. **b** EELS spectra of O-*K* edge, Mn-*L* edge, Fe-*L* edge and the corresponding element contents in the green box area. **c** Contour plots of EELS spectra along the gradient line in the HAADF-STEM image.

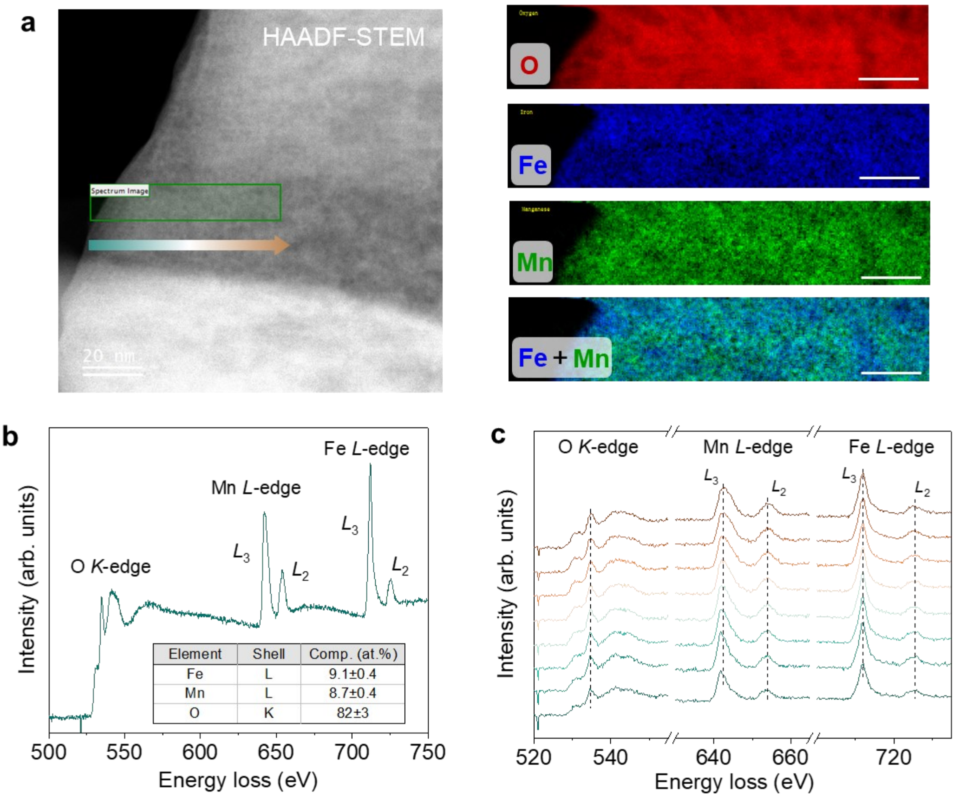

Supplementary Fig. 8 Microstructure and element distribution of (Fe, Mn)$C_2O_4·2H_2O$ precursor. **a** HAADF-STEM image and O, Fe, Mn, and mixed element maps. **b** EELS spectra of O-*K* edge, Mn-*L* edge, Fe-*L* edge and the corresponding element contents in the green box area. **c** Contour plots of EELS spectra along the gradient line in the HAADF-STEM image.

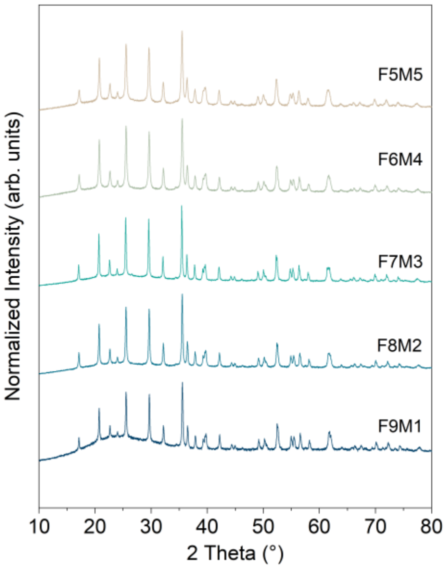

Supplementary Fig. 9 XRD patterns of R-LFMP with different Fe/Mn molar ratios.

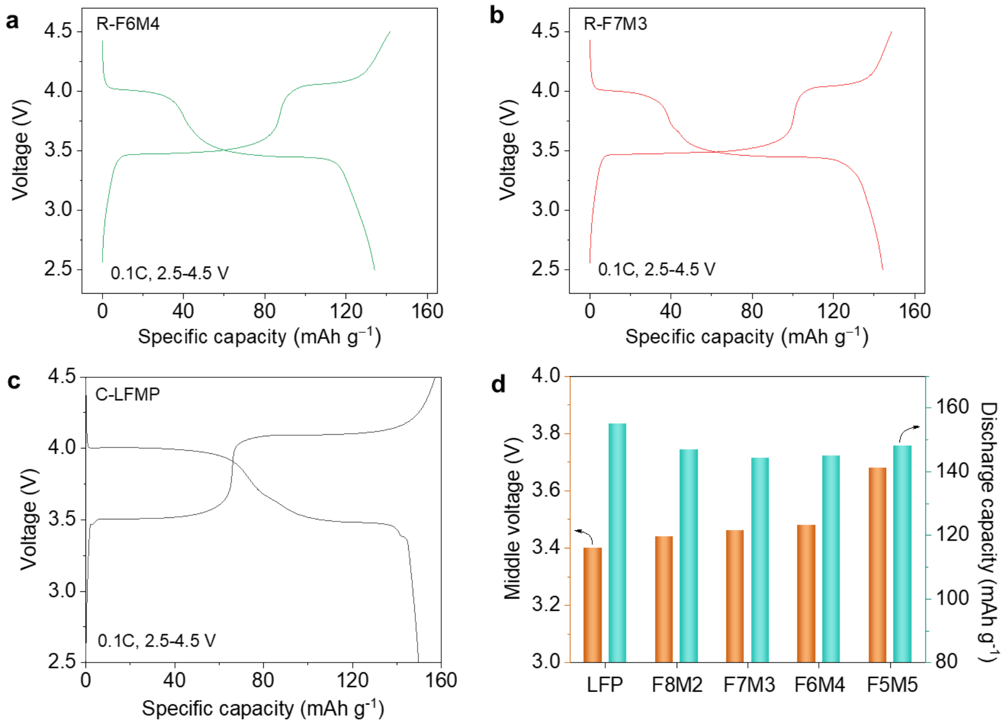

Supplementary Fig. 10 Electrochemical performance of R-LFMP with different Fe/Mn molar ratios. **a-c** Initial charge and discharge curves of **a** R-F6M4, **b** R-F7M3, and **c** C-LFMP. **d** Middle voltages and discharge capacities of LFMP with different Fe/Mn ratios.

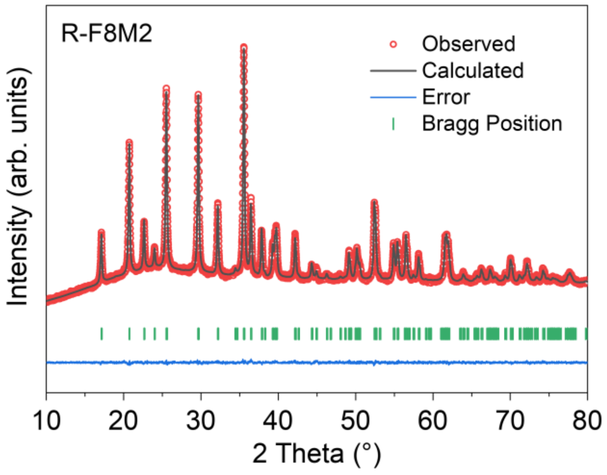

Supplementary Fig. 11 XRD pattern and Rietveld refinement results for R-F8M2.

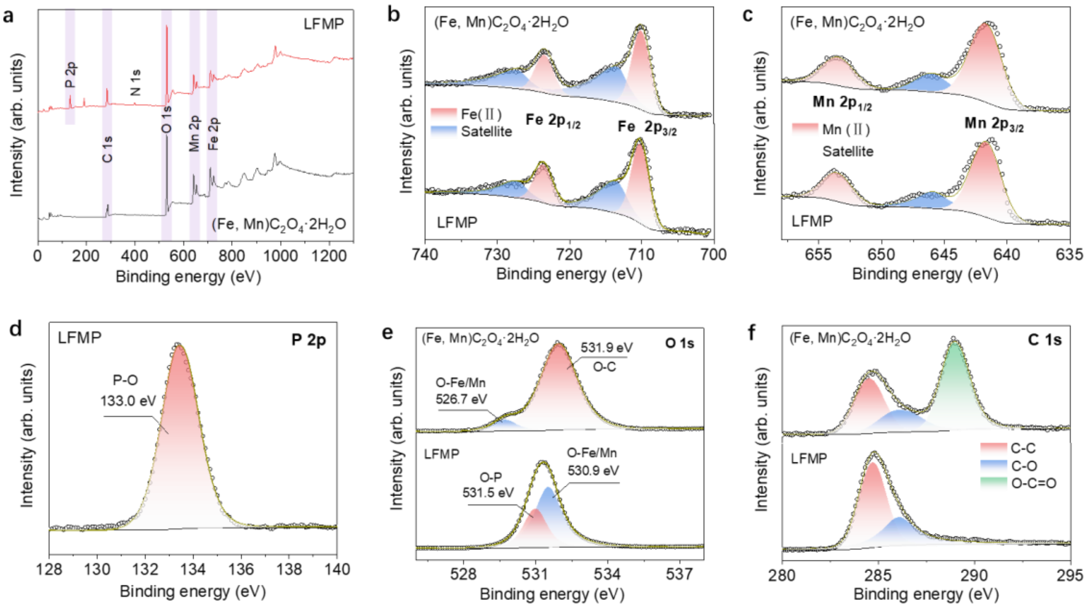

Supplementary Fig. 12 XPS spectra of the precursor and R-F5M5. **a** Survey spectrum. **b** Fe 2*p*. **c** Mn 2*p*. **d** P 2*p*. **e** O 1*s*. **f** C1*s*.

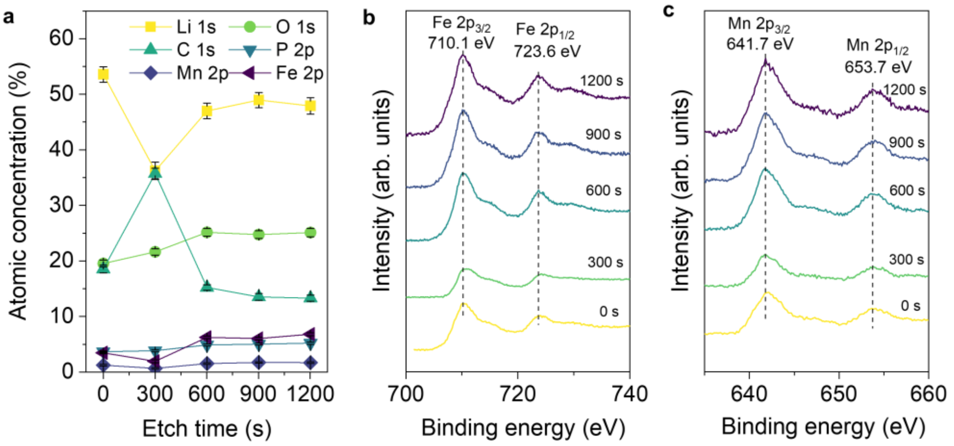

Supplementary Fig. 13 Depth etching XPS results of R-F8M2. **a** Atomic concentrations, **b** Fe 2*p* XPS, **c** Mn 2*p* XPS.

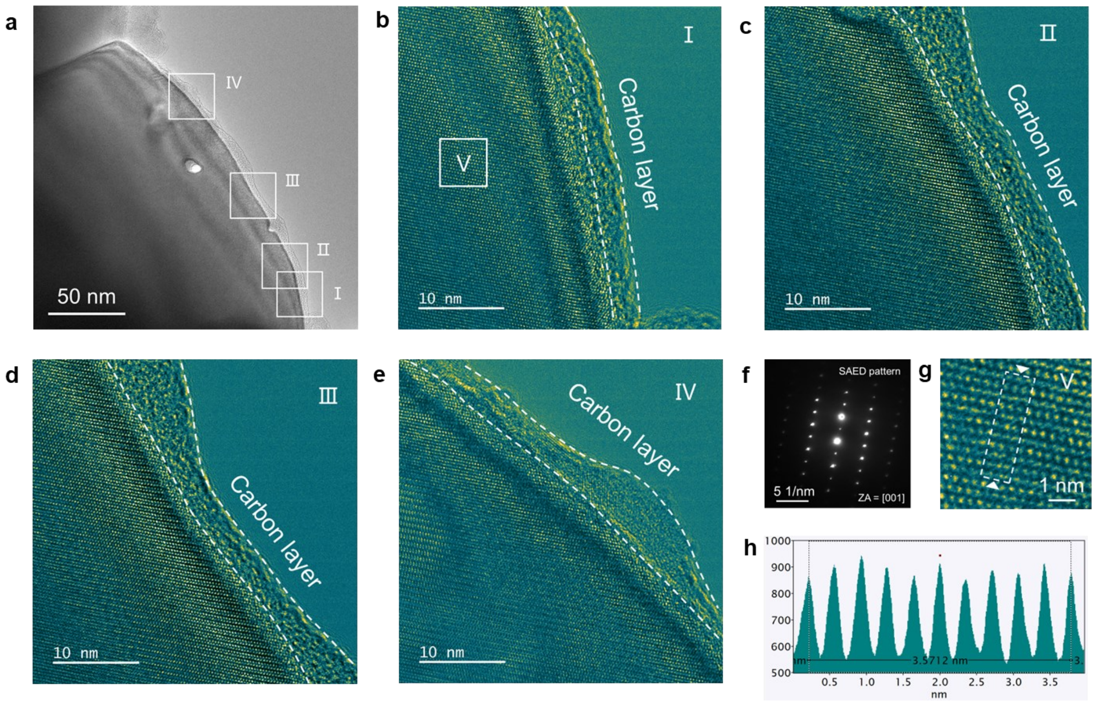

Supplementary Fig. 14 Microstructure characterizations of R-F5M5. **a** TEM image, **b-e** HRTEM images, **f** SAED pattern, **g** Enlarged region in I, **h** Line profiles in V of R-F5M5.

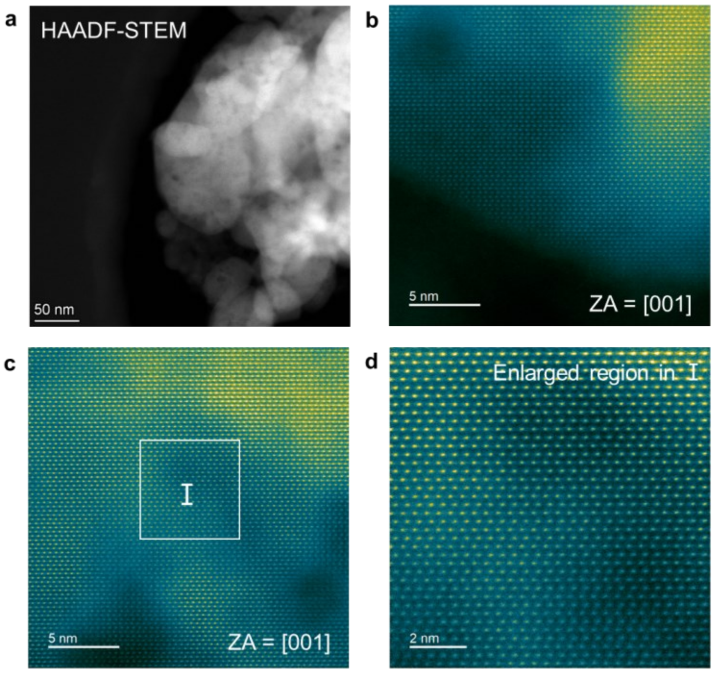

Supplementary Fig. 15 Microstructure characterizations of R-F5M5 at the atomic level. **a** HAADF-STEM image and **b-d** corresponding enlarged regions of R-F5M5.

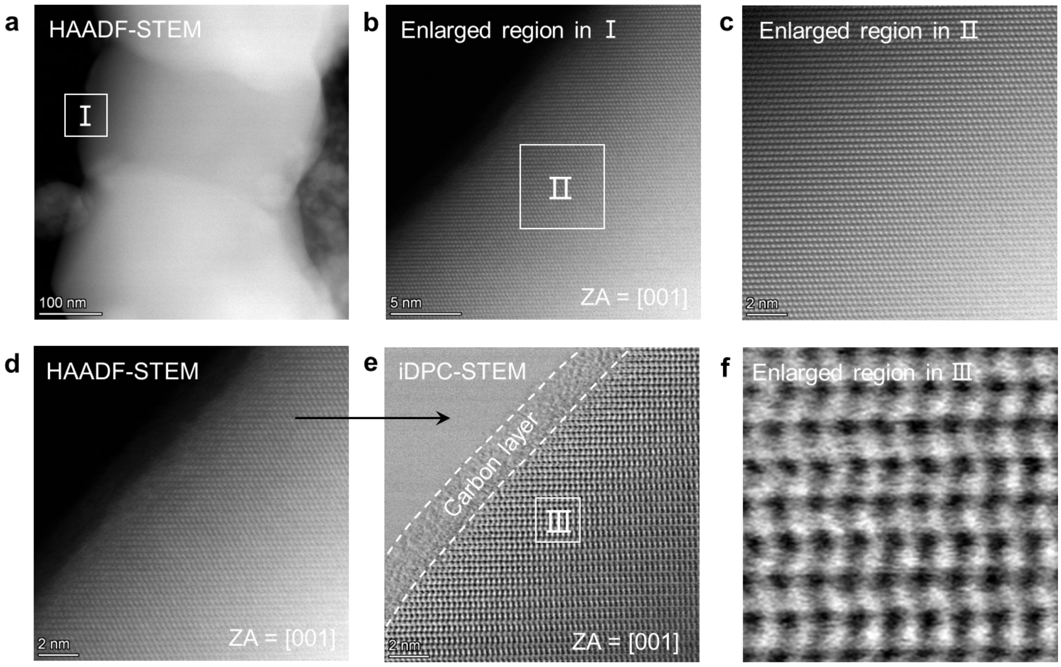

Supplementary Fig. 16 Microstructure characterizations of R-F5M5 at the atomic level. **a** HAADF-STEM image, **b** enlarged area I, **c** enlarged area II, **d** HAADF-STEM image, **e** the corresponding iDPC-STEM image, **f** enlarged region in III.

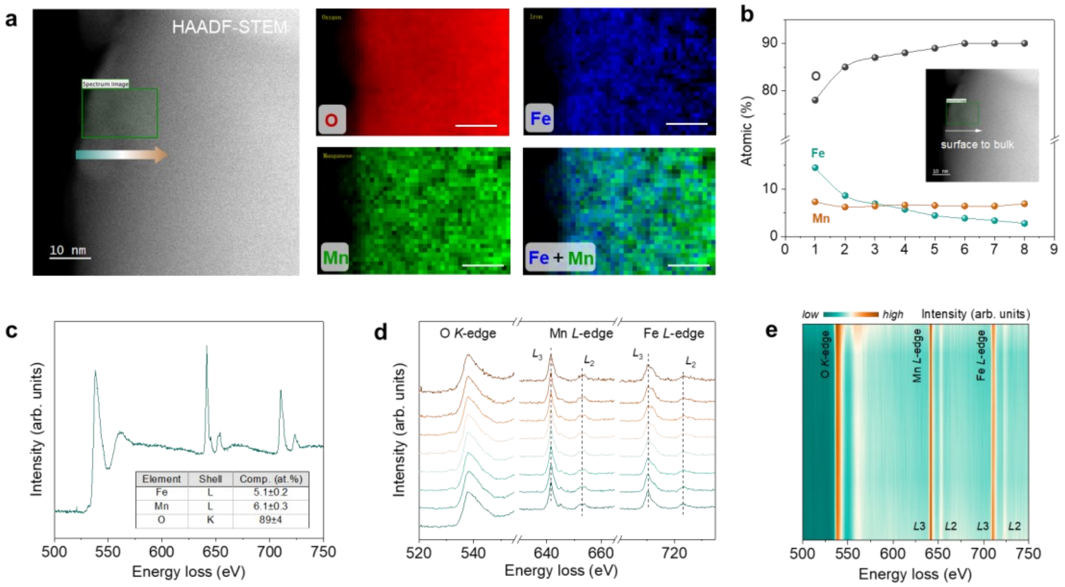

Supplementary Fig. 17 Microstructure and element distribution of R-F5M5. **a** HAADF-STEM image and O, Fe, Mn, and mixed element maps. **b** Atomic concentrations along the gradient line. The inset HAADF-STEM image shows the scanning direction of EELS. **c** EELS spectra of O-*K* edge, Mn-*L* edge, Fe-*L* edge and the corresponding element contents in the green box area. **d** EELS spectra along the gradient line in the HAADF-STEM image. **e** The corresponding contour plots.

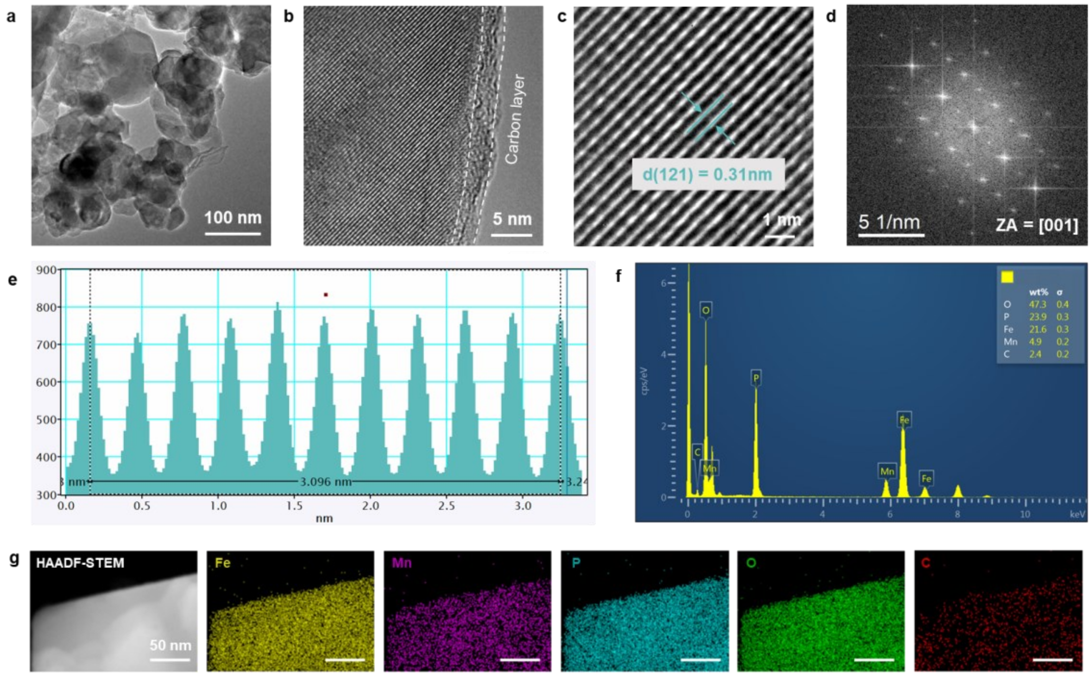

Supplementary Fig. 18 Microstructure characterizations of R-F8M2. **a** TEM image, **b-c** HRTEM images, **d** SAED pattern in **b**, **e** Line profiles in **c**, **f** EDS elemental contents, **g** EDS maps.

The microstructure of R-F8M2 was revealed by TEM and HRTEM images, as displayed in **Supplementary Fig. 18**. The average particle size was about 100 nm and a uniform carbon layer coated on the particle surface with a thickness of 3–4 nm. HAADF-STEM images showed a clear lattice fringe (0.31nm), which was ascribed to the (121) interplanar spacing. Qualitative analysis of EDS results indicated that the elemental contents were consistent with R-F8M2, and Fe, Mn, P, O, and C elements distributed uniformly through the particles.

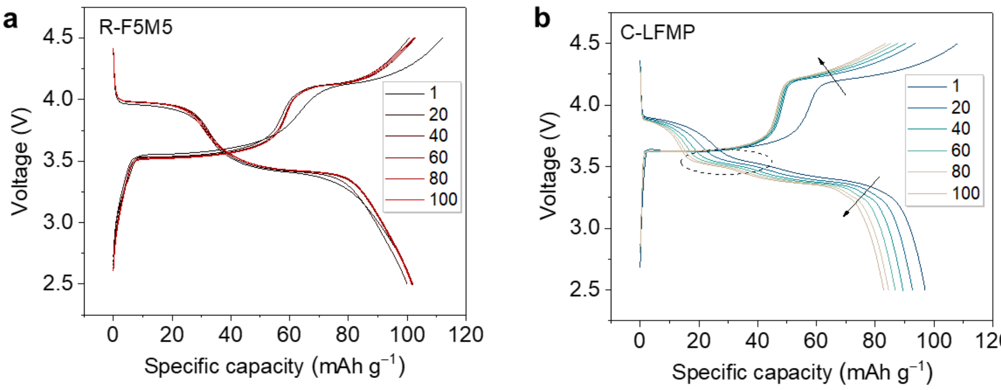

Supplementary Fig. 19 Charge and discharge curves of **a** R-F5M5 and **b** C-LFMP.

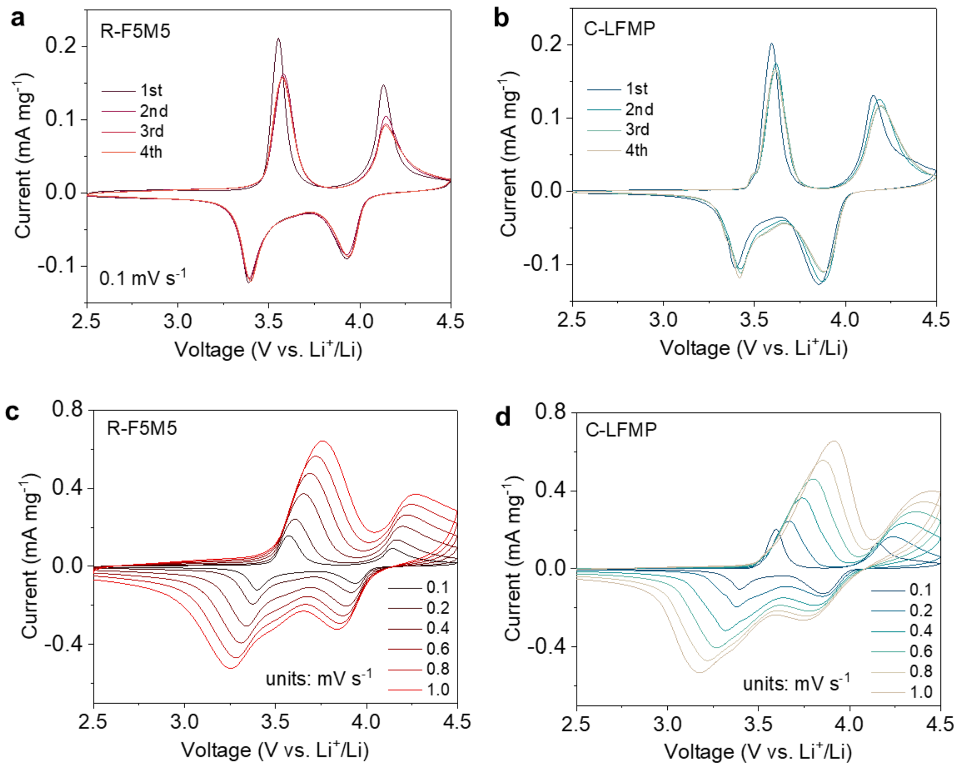

Supplementary Fig. 20 Electrochemical kinetics of R-F5M5 and C-LFMP. **a, b** The initial four CV curves of **a** R-F5M5 and **b** C-LFMP. **c, d** CV curves at different scan rate of **c** R-F5M5 and **d** C-LFMP.

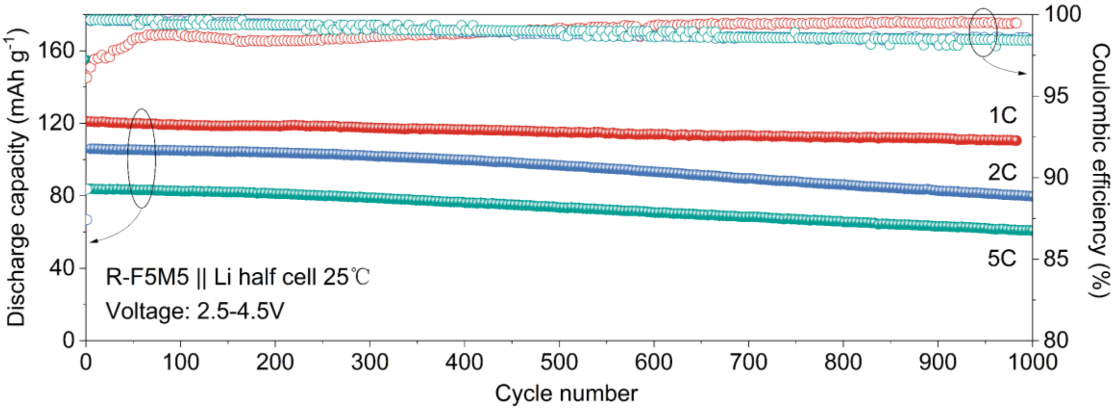

Supplementary Fig. 21 Long-term cycling performance of R-F5M5 at 1C, 2C, and 5C.

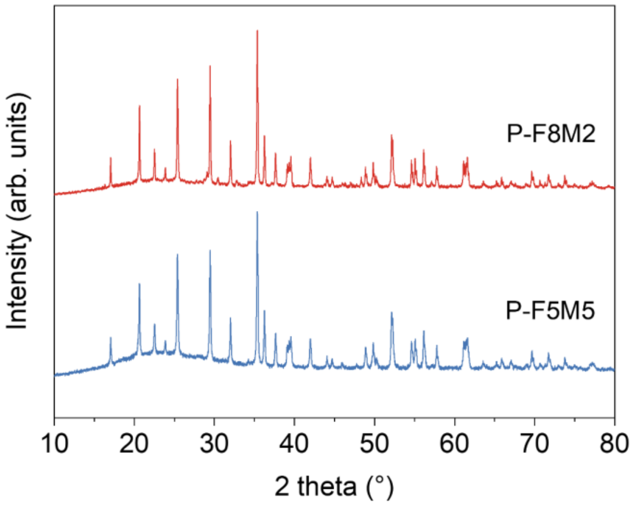

Supplementary Fig. 22 XRD patterns of P-LFMP samples.

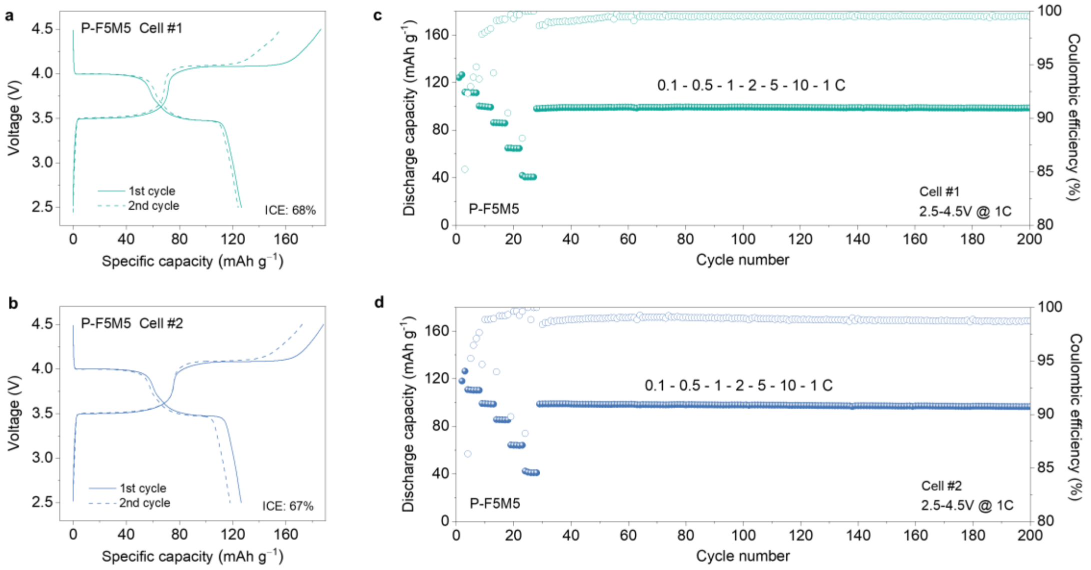

Supplementary Fig. 23 Electrochemical performance of P-F5M5. **a, b** Initial two charge and discharge curves at 0.1C. **c, d** Rate capabilities and cycling performance at 1C rate.

We have synthesized the comparative samples with highly purified raw materials (Chemicals involve FeC_2O_4, $MnC_2O_4 \cdot 2H_2O$, which are analytical grade and purchased from Macklin), as shown in **Supplementary Fig. 22**. All the experimental conditions were the same as for the R-LFMP materials except for the commercial Fe/Mn precursors. Here, we take pristine $LiFe_{0.5}Mn_{0.5}PO_4$ (P-F5M5) as a comparison sample for discussion. As shown in **Supplementary Fig. 23**, P-F5M5 has an initial discharge capacity of 130 mAh g^{-1} with a low initial Coulombic efficiency (ICE) of 68%. As contrast, the initial discharge capacity of R-F5M5 is 152 mAh g^{-1} with ICE of 94%, indicating the superior performance and cyclic reversibility. Regarding the rate performance (0.1-0.5-1-2-5-10C rates), the discharge capacities of 128, 110 and 90 mAh g^{-1} were still retained at 1, 2, and 5C rates for R-F5M5. In contrast, P-F5M5 only have discharge capacities of 100, 86, and 64 mAh g^{-1} under the same rates.

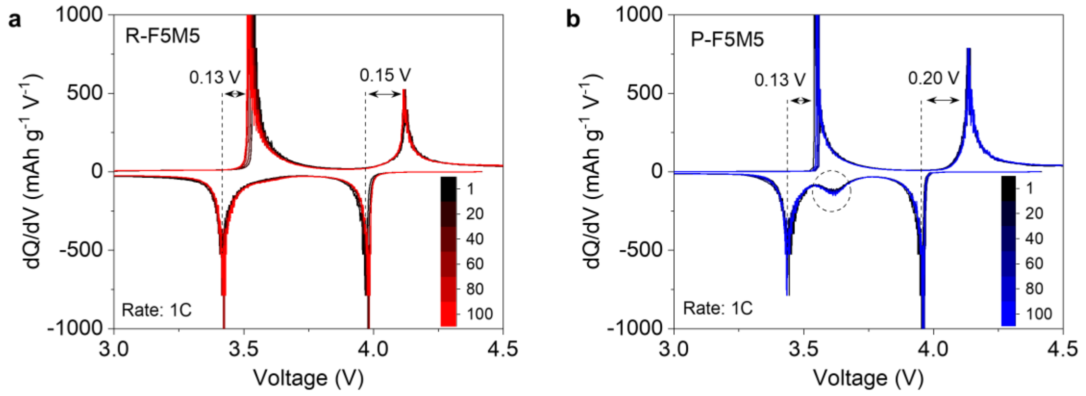

Supplementary Fig. 24 dQ/dV curves from 1st to 100th cycle at 1C rate. **a** R-F5M5, **b** P-F5M5.

To further reveal the reasons for the differences in electrochemical performance, we compared and analyzed the dQ/dV curves. R-F5M5 showed sharp redox peaks with mitigated polarization (0.13 V for $Fe^{+2/+3}$ and 0.15 V for $Mn^{+2/+3}$). In comparison, P-F5M5 had larger polarizations after 100 cycles (0.13 V for $Fe^{+2/+3}$ and 0.20 V for $Mn^{+2/+3}$). An extra peak was observed at 3.6 V during discharge, in agreement with the charge and discharge curves. The same phenomenon was seen in the C-LFMP sample (**Fig. 3g**), ascribed with the structural instability.

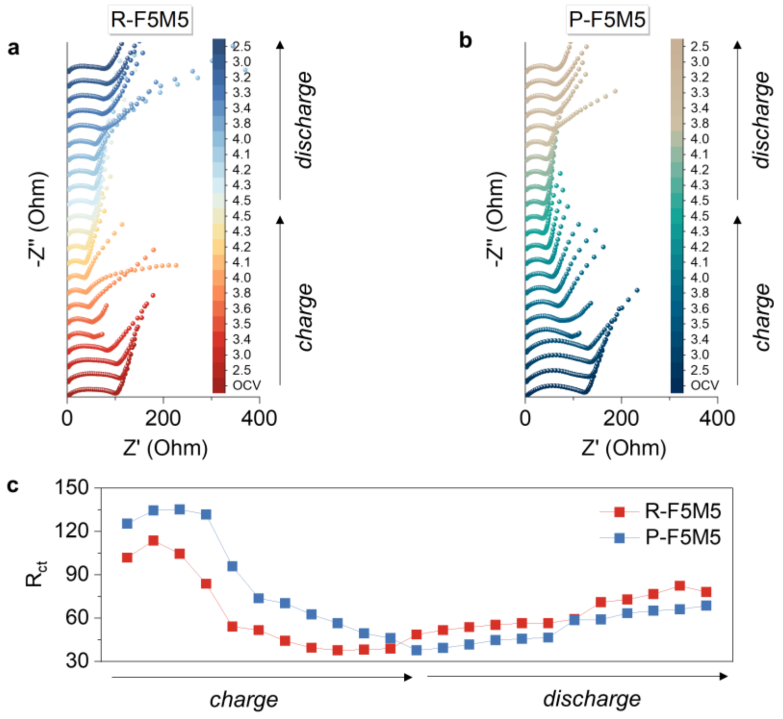

Supplementary Fig. 25 Impedance spectra collected during the first cycle. **a** R-F5M5, **b** P-F5M5. **c** R_{ct} value change trends.

In addition, in-situ EIS measurements were carried out to clarify the change of internal resistance (**Supplementary Fig. 25**). The semicircles at a high frequency are ascribed to the charge transfer contribution from the cathode interfaces. During charging, the charge transfer impedance (R_{ct}) decreased gradually and reached a minimum at 100% state of charge (4.5 V). R-F5M5 had a smaller R_{ct} value compared with P-F5M5, indicating that the electronic and ionic conductivity of the R-F5M5 cathode during delithiation. This also explains why the capacity release and cycling stability of R-F5M5 samples are better that that of P-F5M5. During discharging, the R_{ct} increased slightly and then remained at a steady state. The two samples showed similar results.

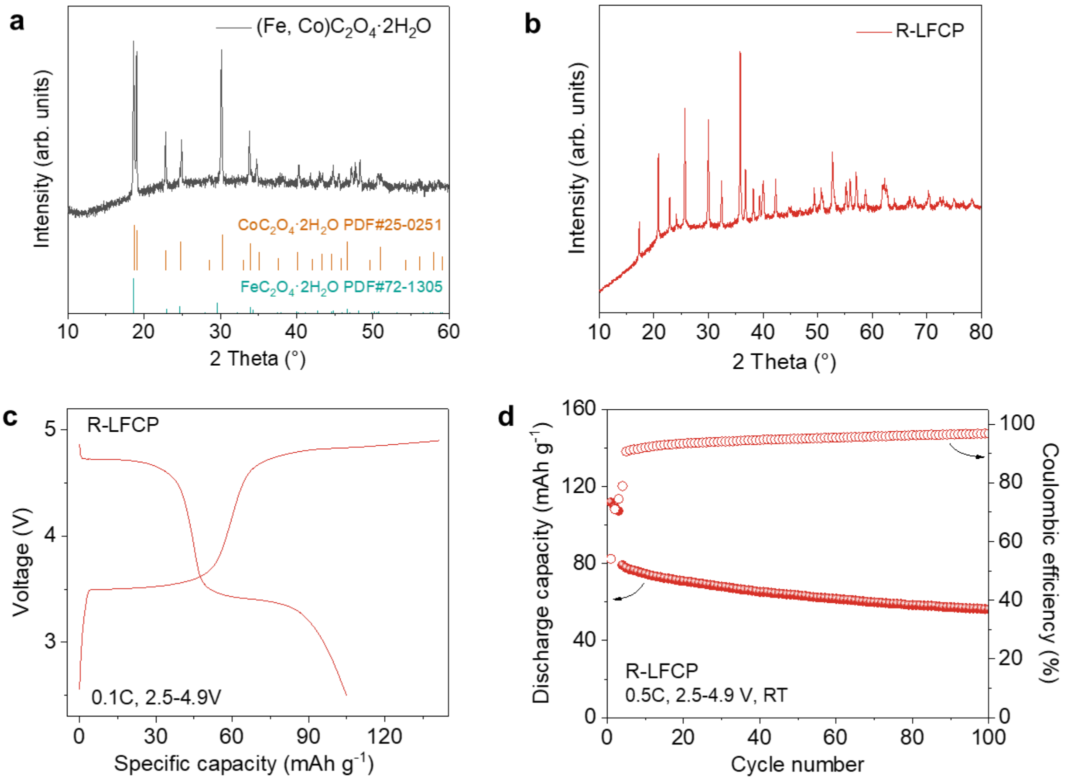

Supplementary Fig. 26 A universal demonstration of the synthesis of LFCP with a higher voltage using our proposed strategy. **a, b** XRD patterns of **a** (Fe, Co)C_2O_4·$2H_2O$ and **b** R-LFCP. **c** Charge and discharge curves. **d** Cycling performance.

In addition to LFMP, our proposed strategy can also be used for synthesizing $LiFe_xCo_{1-x}PO_4$ (LFCP) with a higher voltage. Unfortunately, LFCP has a poor performance because the upper cut-off voltage is beyond the stable range of commercial electrolytes. If there is a suitable electrolyte in the future, the performance of LFCP could be improved.

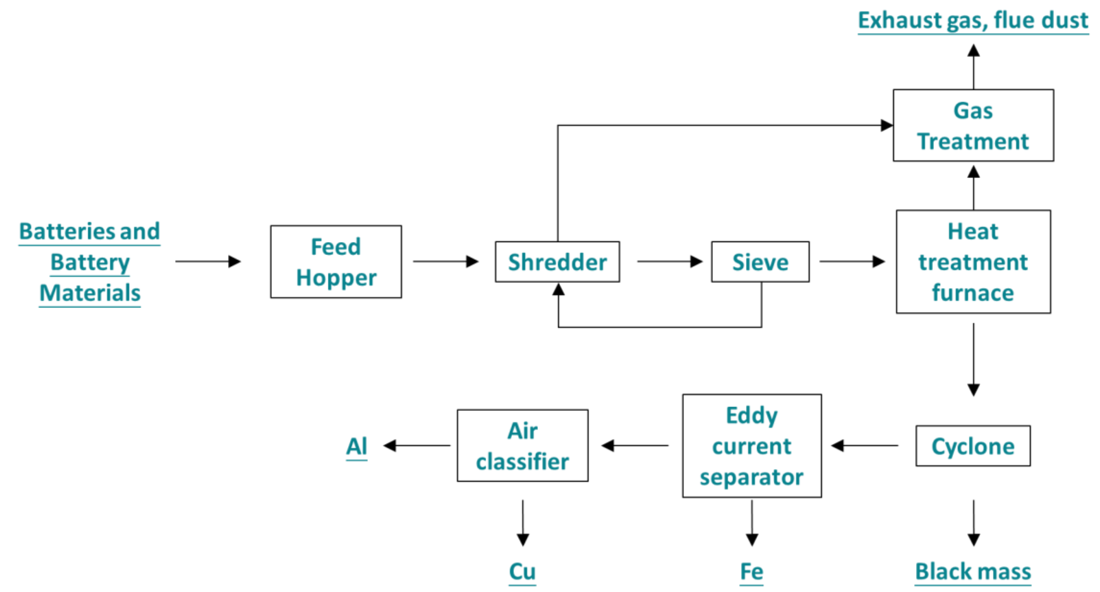

Supplementary Fig. 27 Schematic of the preprocessing of end-of-life batteries. The black mass was used for Pyro, Hydro, Direct, or Upcycle recycling. The Al foil, Cu foil, and other valuable products are sold to compensate for the recycling cost.

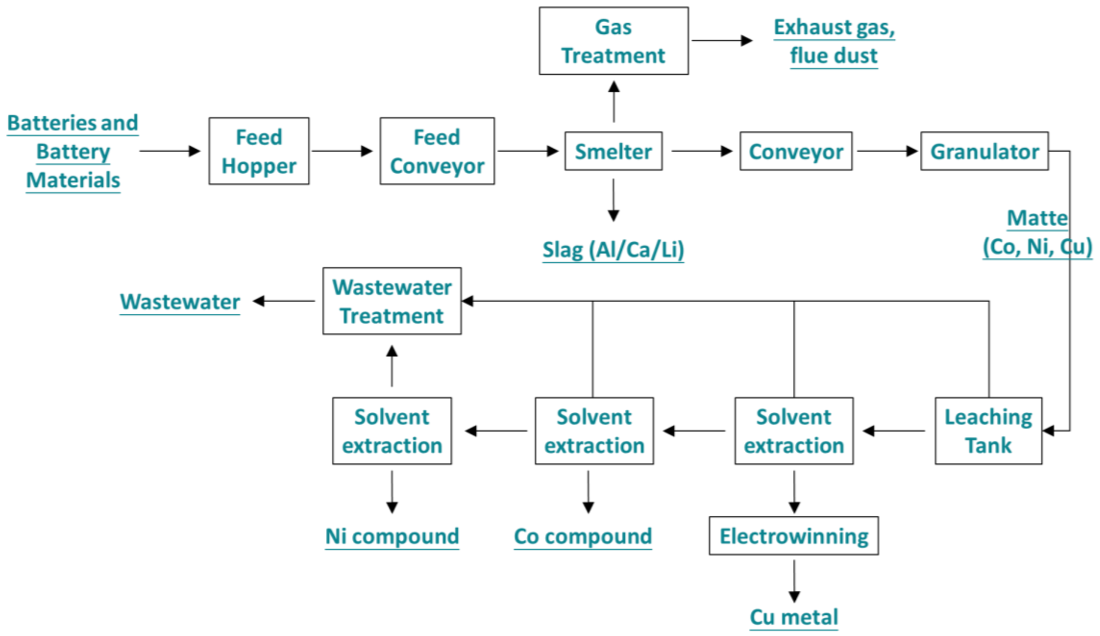

Supplementary Fig. 28 Schematic of Pyro recycling in which preprocessing is not required.

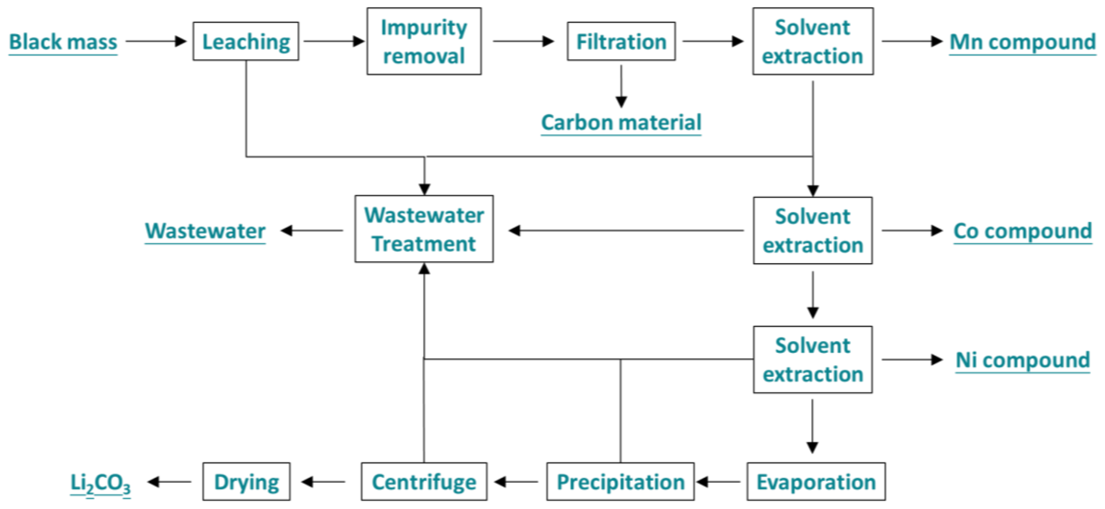

Supplementary Fig. 29 Schematic of Hydro recycling.

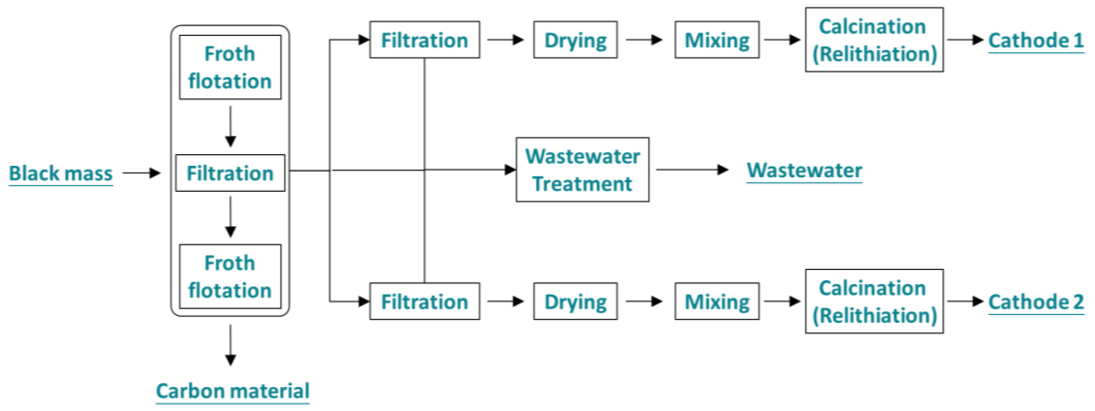

Supplementary Fig. 30 Schematic of direct recycling.

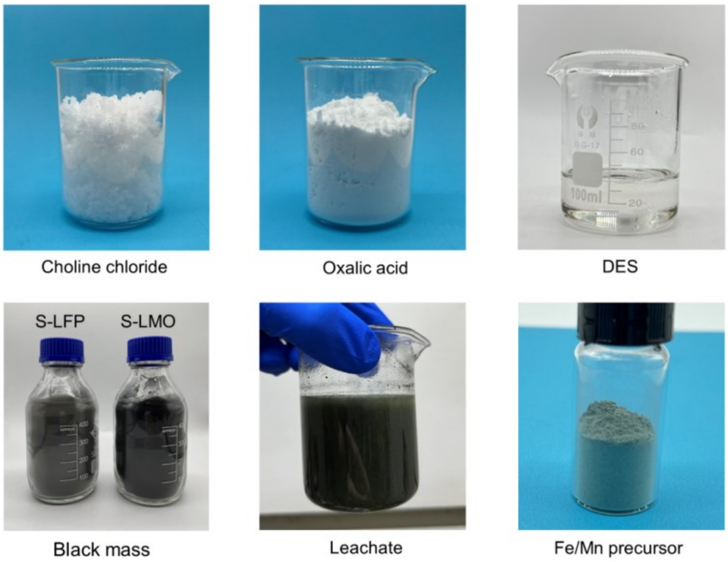

Supplementary Fig. 31 Pictures for scalable experiments in the lab.

We carried out a scalable experiment in the lab (Ten times of the processes in the MS), as depicted in **Supplementary Fig. 31**. Specifically, 56 g choline chloride (ChCl) and 36 g oxalic acid (OA) were mixed in a molar ratio of 1: 1. Then the mixture was heated at 80 °C to form a transparent DES, which was used for leaching the black mass. After which, 1.58 g S-LFP and 0.90 g LMO (Fe: Mn = 1: 1 in a molar ratio) was added into the DES and the mixture was then heated at 110 °C under continuous stirring for 6 h. The subsequent experimental procedures are exactly the same as that in the Methods part. As expected, the experimental phenomenon was consistent with the previous processes. Therefore, the upcycling approach is able to expanding at a gram-grade scale.

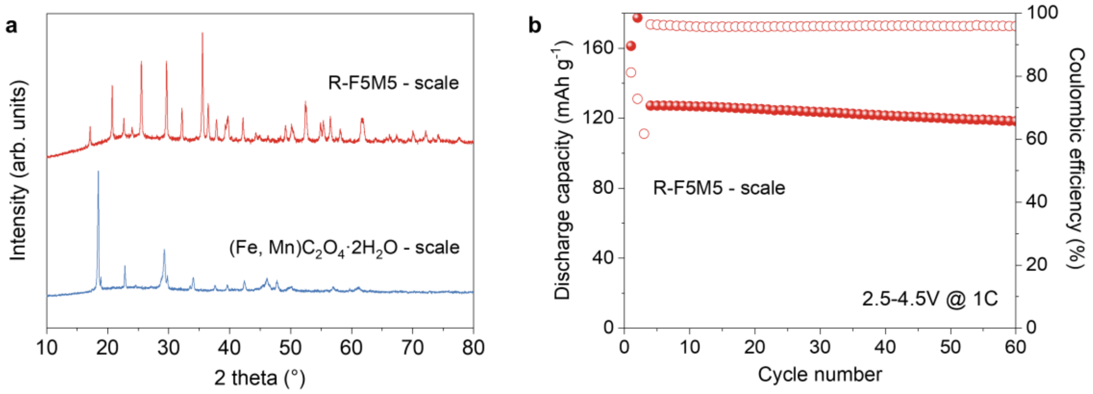

Supplementary Fig. 32 The scalable experiment demonstration results. **a** XRD patterns for the precursor and R-F5M5. **b** Electrochemical performance of R-F5M5-scale sample.

we further synthesized the R-F5M5-scale sample by using the precursor obtained from a scalable experiment, as shown in **Supplementary Fig. 32**. XRD patterns show good phase crystallinity for both (Fe, Mn)$C_2O_4{\cdot}2H_2O$ precursor and R-F5M5-scale sample. In electrochemical properties tests, it delivers a discharge capacity of 130 mAh g^{-1} at 1C rate, which was a very satisfying result.

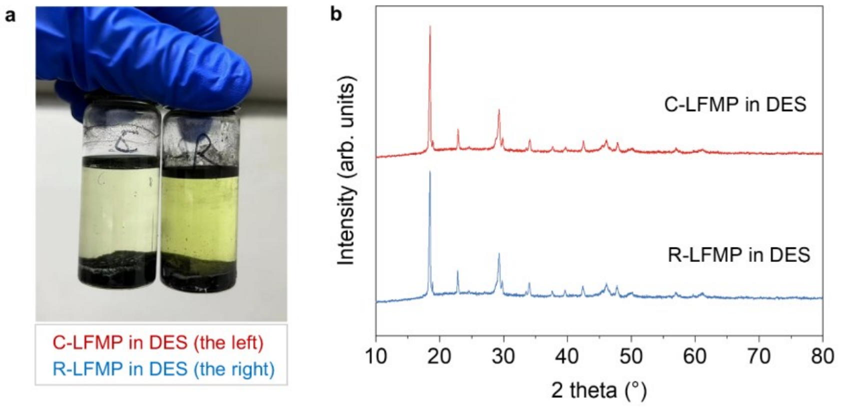

Supplementary Fig. 33 The feasibility verification for recycling LFMP using our proposed strategy. **a** Picture for C-LFMP and R-LFMP dissolved in the DES. **b** XRD patterns for the obtained precursors.

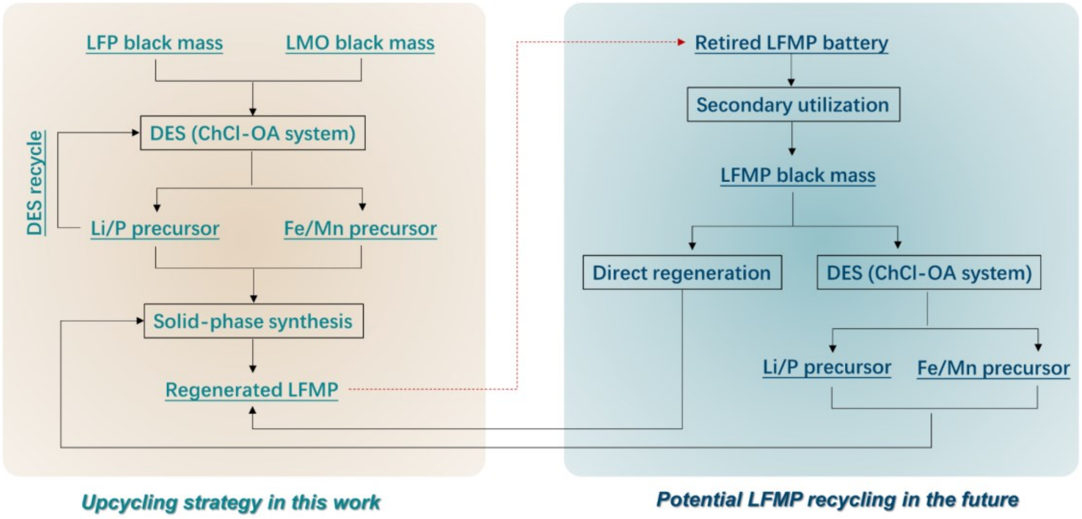

Supplementary Fig. 34 Schematic for the upcycling strategy in this work and the potential LFMP recycling in the future.

We have expanded the scope of practical applications of the upcycling strategy, that is, a potential recycling approach for LFMP was proposed and verified by experiments. LFMP has the same olivine type crystal structure as LFP and can be dissolved by the DES. As shown in **Supplementary Fig. 33**, here we used the commercial LFMP (C-LFMP) and R-LFMP as the raw materials, respectively. The same target product was formed, that is, the Fe/Mn oxalate precursor, suggesting that the upcycling approach in this work is also applicative to process LFMP cathodes. A sustainable recycling system, involving the upcycling strategy in this work and potential LFMP recycling in the future, is summarized in **Supplementary Fig. 34**.

Supplementary Table 1 The leaching rates of Li/P/Fe/Mn based on ICP results.

	**Li**	**P**	**Fe**	**Mn**
Filtrate-1	0.991	0.957	0.113	0.206
Filtrate-2	0.952	0.973	0.074	0.141
Filtrate-3	0.959	0.981	0.126	0.185

Note: 1 mmol S-LFP and 0.5 mmol S-LMO were used to calculate the leaching rate. Three experiments were used to get the average value. The leaching rates were calculated based the filtrate after removing (Fe, Mn)$C_2O_4 \cdot 2H_2O$ precipitation.

Supplementary Table 2 The element contents of R-LFMP based on ICP results.

	**Molar ratio**		
	**Fe/(Fe+Mn)**	**Li/(Fe+Mn)**	**Li/P**
R-$LiFe_{0.5}Mn_{0.5}PO_4$ (F5M5)	0.51	1.09	0.95
R-$LiFe_{0.6}Mn_{0.4}PO_4$ (F6M4)	0.62	0.95	1.05
R-$LiFe_{0.7}Mn_{0.3}PO_4$ (F7M3)	0.69	1.12	0.96
R-$LiFe_{0.8}Mn_{0.2}PO_4$ (F8M2)	0.82	1.10	0.95

Supplementary Table 3 The XRD refinement results for R-F5M5.

**$LiFe_{0.5}Mn_{0.5}PO_4$** (Space Group *Pnma*)						
	**Atom**	**x**	**y**	**z**	**Occ.**	**Biso.**
**Atomic Occupancies**	Li	0.0000	0.0000	0.0000	1.0	2.5732(3)
	Fe	0.2815(1)	0.2500	0.9737(3)	0.5	1.0946(4)
	Mn	0.2815(1)	0.2500	0.9737(3)	0.5	
	P	0.0950(1)	0.2500	0.4197(4)	1.0	2.3453(1)
	O_1	0.0880(1)	0.2500	0.7435(3)	1.0	1.6706(2)
	O_2	0.4505(6)	0.2500	0.2350(5)	1.0	1.3968(9)
	O_3	0.1579(3)	0.0490(7)	0.2779(1)	1.0	1.4235(7)
**Lattice Parameters**	***a*** **/ Å**	***b*** **/ Å**	***c*** **/ Å**	**V / Å^3**		
	10.3935(2)	6.0541(7)	4.7188(8)	296.83(1)		
**Agreement Factors**						
**χ^2** 1.79%	**R_p** 1.76%	**R_{wp}** 2.68%				

Supplementary Table 4 The XRD refinement results for R-F8M2.

**$LiMn_{0.2}Fe_{0.8}PO_4$** (Space Group Pnma)						
	**Atom**	**x**	**y**	**z**	**Occ.**	**Biso.**
**Atomic Occupancies**	Li	0.0000	0.0000	0.0000	1.0	0.6226(2)
	Fe	0.2820(1)	0.2500	0.9731(9)	0.8	2.0686(8)
	Mn	0.2820(1)	0.2500	0.9731(9)	0.2	
	P	0.0963(1)	0.2500	0.4261(1)	1.0	2.1677(4)
	O_1	0.0874(1)	0.2500	0.7460(2)	1.0	2.2195(2)
	O_2	0.4543(1)	0.2500	0.2222(2)	1.0	1.8578(8)
	O_3	0.1591(5)	0.0519(4)	0.2798(8)	1.0	1.9197(5)
**Lattice Parameters**	***a*** **/ Å**	***b*** **/ Å**	***c*** **/ Å**	**V / Å^3**		
	10.3415(3)	6.0208(5)	4.7021(9)	296.78(2)		
**Agreement Factors**						
**χ^2** 1.41%	**R_p** 1.27%	**R_{wp}** 1.02%				

Supplementary Table 5 The element contents of R-LFCP based on ICP results.

	Molar ratio		
	**Fe/(Fe+Co)**	**Li/(Fe+Co)**	**Li/P**
R-$LiFe_xCo_{1-x}PO_4$ (LFCP)	0.437	1.033	1.024

Supplementary Table 6 Preprocessing throughput information.

	Preprocessing throughput		
	Type	tonne/yr	Geographic location
S-LFP	End-of-life battery: cell	10,000	China
S-LMO	End-of-life battery: cell	10,000	

Note: 13,517 tonnes black mass was got after preprocessing, including 36.0% LMO, 32.1% LFP, 29.3% graphite, 1.4% carbon black, 0.1% binder, 0.7% copper, 0.4% aluminum. In Pyro recycling, preprocessing is not required.

Supplementary Table 7 The usage and cost of consumed chemicals.

	**Material**	**Usage (kg)**	**Unit price ($ per kg)**
**Pyro**	Limestone	0.09	0.13
	Sand	0.14	0.05
	Sulfuric acid	0.14	0.08
	Lime	0.08	0.13
**Hydro**	Sulfuric acid	0.97	0.08
	Hydrogen Peroxide	0.07	1.46
	Sodium Hydroxide	0.64	0.45
	Soda Ash	0.21	0.14
	Lime	0.001	0.13
**Direct**	Lithium hydroxide	0.03	34.89
**Upcycle**	Lithium hydroxide	0.09	34.89
	Choline chloride	5.70	2.77
	Oxalic acid	2.88	0.53

Note: The unite price is obtained from Everbatt 2023 database.

Supplementary Table 8 The quantities and values of recycled materials.

	Products	Quantity (kg)	Unit price ($ per kg)
**Pyro**	Copper metal	0.09	7.37
**Hydro**	Lithium carbonate	0.133	8.57
	Mn^{2+} in product	0.214	3.15
	Graphite	0.264	0.20
	Copper	0.006	7.11
	Aluminum	0.004	1.12
**Direct**	LFP	0.289	10.00
	LMO	0.324	9.00
	Graphite	0.264	0.20
	Copper	0.006	7.11
	Aluminum	0.004	1.12
**Upcycle**	LFMP	0.580	12.00
	Lithium carbonate	0.107	8.57
	Mn^{2+} in product	0.133	3.15
	Graphite	0.264	0.20
	Copper	0.006	7.11
	Aluminum	0.004	1.12

Note: The value of recycled materials was obtained from the Everbatt 2023 database. The market price of the LFMP material is unknown because it is not commercialized. In this TEA analysis, we assumed that the price of LFMP is 1.2 times that of LFP.

Supplementary Table 9 Recycling cost ($ per kg feedstock) of different battery recycling technologies.

	**Pyro**	**Hydro**	**Direct**	**Upcycle**
Materials	0.04	0.37	0.72	2.16
Utilities	0.03	0.06	0.21	0.20
Other variable costs	0.01	0.02	0.02	0.05
Labor	0.02	0.03	0.03	0.03
Maintenance	0.15	0.10	0.12	0.06
Plant overhead	0.03	0.06	0.09	0.12
Other fixed costs	0.20	0.13	0.17	0.11
Annualized capital cost	0.94	0.61	0.74	0.28
Feedstock payment	(1.00)	(1.00)	(1.00)	(1.00)

Supplementary Table 10 Recycling revenue ($ per kg feedstock) of different battery recycling technologies.

	**Pyro**	**Hydro**	**Direct**	**Upcycle**
R-LFP	0	0	2.89	0
R-LMO	0	0	2.91	0
R-LFMP	0	0	0	6.96
Mn^{2+} in product	0	0.67	0	0.34
Lithium carbonate	0	1.14	0	1.14
Graphite	0	0.05	0.05	0.05
Copper	0.65	0.04	0.04	0.07
Aluminum	0	0.004	0.004	0.004

Supplementary Table 11 Recycling profit ($ per kg feedstock) of different battery recycling technologies.

	**Pyro**	**Hydro**	**Direct**	**Upcycle**
Cost	0.42	0.37	1.10	2.01
Revenue	0.65	1.95	4.64	6.44
Profit	0.23	1.58	3.54	4.43

Supplementary Table 12 GHG emission (g per kg feedstock) and total energy consumption (MJ per kg feedstock) of different battery recycling technologies.

	**Pyro**	**Hydro**	**Direct**	**Upcycle**
GHGs	1455	1814	2438	495
Total energy	4.208	23.005	28.030	19.604

References

1. Zheng, C.-H. & Fang, D.-L. Preparation of ultra-fine cobalt–nickel manganite powders and ceramics derived from mixed oxalate. *Mater. Res. Bull.* **43**, 1877-1882 (2008).

2. Vidyasagar, K., Gopalakrishnan, J. & Rao, C. N. R. A convenient route for the synthesis of complex metal-oxides employing solid-solution precursors. *Inorg. Chem.* **23**, 1206-1210 (1984).

3. Thompson, D. L., Pateli, I. M., Lei, C., Jarvis, A., Abbott, A. P. & Hartley, J. M. Separation of nickel from cobalt and manganese in lithium ion batteries using deep eutectic solvents. *Green Chem.* **24**, 4877-4886 (2022).
