# Supplementary material for: Sustainable upcycling of mixed spent cathodes to a high-voltage polyanionic cathode material
Source: Nat Commun. 2024 May 14;15:4086. doi: 10.1038/s41467-024-48181-9 (PMC11094161; doi:10.1038/s41467-024-48181-9)
Supplement: Supplementary file 3 — Reporting Summary [file 41467_2024_48181_MOESM3_ESM.pdf]

nature portfolio

Corresponding author(s): Junxiong Wang, Guangmin Zhou; Hui-Ming Cheng

Last updated by author(s): Mar 29, 2024

Reporting Summary

Nature Portfolio wishes to improve the reproducibility of the work that we publish. This form provides structure for consistency and transparency in reporting. For further information on Nature Portfolio policies, see our Editorial Policies and the Editorial Policy Checklist.

Statistics

For all statistical analyses, confirm that the following items are present in the figure legend, table legend, main text, or Methods section.

n/a	Confirmed	
☒	☐	The exact sample size (*n*) for each experimental group/condition, given as a discrete number and unit of measurement
☒	☐	A statement on whether measurements were taken from distinct samples or whether the same sample was measured repeatedly
☒	☐	The statistical test(s) used AND whether they are one- or two-sided<br>*Only common tests should be described solely by name; describe more complex techniques in the Methods section.*
☒	☐	A description of all covariates tested
☒	☐	A description of any assumptions or corrections, such as tests of normality and adjustment for multiple comparisons
☒	☐	A full description of the statistical parameters including central tendency (e.g. means) or other basic estimates (e.g. regression coefficient) AND variation (e.g. standard deviation) or associated estimates of uncertainty (e.g. confidence intervals)
☒	☐	For null hypothesis testing, the test statistic (e.g. *F*, *t*, *r*) with confidence intervals, effect sizes, degrees of freedom and *P* value noted<br>*Give P values as exact values whenever suitable.*
☒	☐	For Bayesian analysis, information on the choice of priors and Markov chain Monte Carlo settings
☒	☐	For hierarchical and complex designs, identification of the appropriate level for tests and full reporting of outcomes
☒	☐	Estimates of effect sizes (e.g. Cohen's *d*, Pearson's *r*), indicating how they were calculated

Our web collection on statistics for biologists contains articles on many of the points above.

Software and code

Policy information about availability of computer code

Data collection	None
Data analysis	None

For manuscripts utilizing custom algorithms or software that are central to the research but not yet described in published literature, software must be made available to editors and reviewers. We strongly encourage code deposition in a community repository (e.g. GitHub). See the Nature Portfolio guidelines for submitting code & software for further information.

Data

Policy information about availability of data

All manuscripts must include a data availability statement. This statement should provide the following information, where applicable:

- Accession codes, unique identifiers, or web links for publicly available datasets
- A description of any restrictions on data availability
- For clinical datasets or third party data, please ensure that the statement adheres to our policy

The datasets generated during and/or analysed during the current study are available from the corresponding author on reasonable request. Source data are provided with this paper.

Research involving human participants, their data, or biological material

Policy information about studies with human participants or human data. See also policy information about sex, gender (identity/presentation), and sexual orientation and race, ethnicity and racism.

Reporting on sex and gender	This is not relevant to this study.
Reporting on race, ethnicity, or other socially relevant groupings	This is not relevant to this study.
Population characteristics	This is not relevant to this study.
Recruitment	This is not relevant to this study.
Ethics oversight	This is not relevant to this study.

Note that full information on the approval of the study protocol must also be provided in the manuscript.

Field-specific reporting

Please select the one below that is the best fit for your research. If you are not sure, read the appropriate sections before making your selection.

☐ Life sciences ☐ Behavioural & social sciences ☒ Ecological, evolutionary & environmental sciences

For a reference copy of the document with all sections, see nature.com/documents/nr-reporting-summary-flat.pdf

Ecological, evolutionary & environmental sciences study design

All studies must disclose on these points even when the disclosure is negative.

Study description	An upcycling strategy was proposed for mixed LiFePO4 and Mn-rich cathodes in spent Li-ion batteries. By structural design and transition metal replacement, the regenerated high-voltage polyanionic cathode materials show an increased energy density and economic value. The green deep eutectic solvent used can be recycled, while providing a sustainable approach for the value-added utilization of waste battery materials.
Research sample	spent LiFePO4 and LiMn2O4 cathodes, the regenerated LiFexMn1-xPO4 cathode materials
Sampling strategy	Each testing group contains three parallel samples to ensure the reliability and repeatability of the test battery performance.
Data collection	All battery test data are automatically recorded by the battery test system.
Timing and spatial scale	The start and end of the battery test are based on the voltage. The charge and discharge test will start when the voltage reaches the set value of charge and discharge cut-off voltage, respectively. The whole cycle test will end when the battery capacity decays to a certain extent.
Data exclusions	No data was excluded.
Reproducibility	Each group of test cells has at least three parallel samples to ensure reproducibility of experimental results.
Randomization	This is not relevant to this study. The selection of experimental samples and test conditions are strictly controlled and kept consistent, so there is no randomization in this study.
Blinding	This is not relevant to this study. There are many researches on recycling of battery materials, and there are many referable results, which are very clear. The results of this paper are clear, and all the details are described carefully, so there is no problem of blinding.

Did the study involve field work? ☐ Yes ☒ No

Reporting for specific materials, systems and methods

We require information from authors about some types of materials, experimental systems and methods used in many studies. Here, indicate whether each material, system or method listed is relevant to your study. If you are not sure if a list item applies to your research, read the appropriate section before selecting a response.

Materials & experimental systems

n/a	Involved in the study
☒	☐ Antibodies
☒	☐ Eukaryotic cell lines
☒	☐ Palaeontology and archaeology
☒	☐ Animals and other organisms
☒	☐ Clinical data
☒	☐ Dual use research of concern
☒	☐ Plants

Methods

n/a	Involved in the study
☒	☐ ChIP-seq
☒	☐ Flow cytometry
☒	☐ MRI-based neuroimaging

Plants

Seed stocks	This is not relevant to this study.
Novel plant genotypes	This is not relevant to this study.
Authentication	This is not relevant to this study.
